# Case Report: Report of Infective Endocarditis Caused by *Abiotrophia defectiva* and Literature Review

**DOI:** 10.3389/fped.2022.894049

**Published:** 2022-07-06

**Authors:** Yanqiang Du, Zhan Zhang, Chao Chen, Han Xia, Hua Zhang, Zhangyan Guo, Yi Wang

**Affiliations:** ^1^Department of Pediatric Intensive Care Unit of Xi’an Children’s Hospital, National Children’s Regional Medical Center (Northwest), Children’s Hospital, Xi’an Jiaotong University, Xi’an, China; ^2^Department of Ultrasound of Xi’an Children’s Hospital, National Children’s Regional Medical Center (Northwest), Children’s Hospital, Xi’an Jiaotong University, Xi’an, China; ^3^Imaging Department of Xi’an Children’s Hospital, National Children’s Regional Medical Center (Northwest), Children’s Hospital, Xi’an Jiaotong University, Xi’an, China; ^4^Hugobiotech Co., Ltd., Beijing, China

**Keywords:** children, *Abiotrophia defectiva*, infective endocarditis, metagenomic next-generation sequencing (mNGS), pathogen diagnosis

## Abstract

**Objective:**

To report the clinical features of the first child with infective endocarditis (IE) caused by *Abiotrophia defectiva* in mainland China and to raise awareness of the disease.

**Methods:**

The clinical data of a child with IE caused by *A. defectiva* admitted to Xi’an Children’s Hospital in July 2021 were collected, and the relevant literature was reviewed.

**Results:**

The child was a female, 8 years old, admitted with fever for 4 days and right-sided limb weakness for 3 days. The illness started with suppurative tonsillitis, followed by headache, fatigue, right-sided mouth, slurred speech, right limb weakness, and unstable holding. Transthoracic echocardiography showed that the mitral valve vegetation was formed and vegetation could also be seen at the entrance of the pulmonary vein at the posterior wall of the left atrium. Cranial contrast-enhanced MRI + magnetic resonance angiography showed multiple intracranial pseudoaneurysm formation and pontine infarction. After *A. defectiva* was detected by metagenomic next-generation sequencing (mNGS) in cerebrospinal fluid and blood detected, the infection was controlled by anti-infective treatment with meropenem and vancomycin. On the 36th day after admission, due to severe headache and slurred speech, the head CT showed hemorrhage of right parietal pseudoaneurysm and cerebral sickle hernia, and right temporo-occipital hematoma evacuation, cerebrovascular malformation resection, and cranial decompression were performed immediately. After the surgery, her speech ability gradually recovered, the muscle strength of her left upper limb was about grade III, while the muscle strength of the rest of the limbs was normal. After a total of 60 days of hospitalization, her family requested to be discharged.

**Conclusion:**

This pediatric patient is the first case of childhood IE caused by *A. defectiva* in mainland China, and the first time in the world that *A. defectiva* was detected by mNGS in patients with IE.

## Introduction

*Abiotrophia defectiva* is a facultative anaerobic gram-positive streptococcus (1–3) which is a normal flora of human oral, upper respiratory, urogenital, and intestinal tracts. In 1961, Frenkel and Hirsch (4) defined a virulent streptococcus with “satellite” growth around other bacterial colonies as nutritionally variant *Streptococci*. In 2000, Collins and Lawson (3) classified nutritionally variant *Streptococci* into *Abiotrophia* and *Granulicatella* by 16S rRNA gene sequence, while *Abiotrophia* has only one species, *A. defectiva*.

When the body is immunocompromised, it becomes an opportunistic pathogen and may cause infective endocarditis (IE) (4), an inflammatory endocardial lesion caused by a variety of pathogens, usually streptococcal and staphylococcal infections (5). IE caused by *A. defectiva* is clinically uncommon, accounting for 5–6% of all patients with IE (5), but it has a high mortality rate and many complications. With the progression of the disease, complications such as septic arthritis are observed.

Only a few pediatric cases have been reported internationally (6), and there are no clinical studies with large sample sizes. A search of PubMed, China National Knowledge Infrastructure, Wanfang Medical Online, and other databases revealed that one pediatric case of IE caused by *A. defectiva* was reported in Taiwan in 2002 (7). As far as we know, the first pediatric case of IE caused by *A. defectiva* in mainland China is reported as follows.

## Case Presentation

**FIGURE 1:**
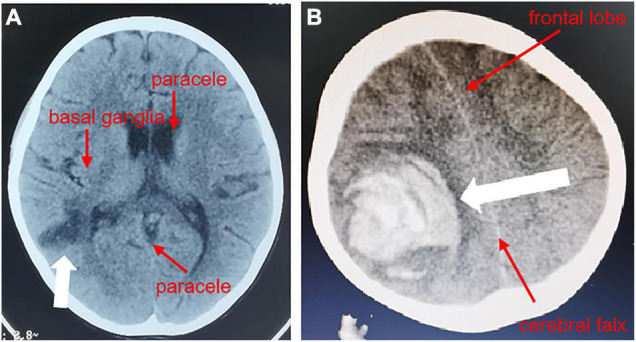
Head CT of the pediatric patient. In the CT results of the head from another hospital, a transverse plane through the inferior portion of the parieto-occipital sulcus showed a hypodense shadow next to the posterior horn of the right ventricle **(A)**, and on the 36th day of hospitalization, a transverse plane through the center of the hemioval showed a right parietal hematoma with herniation of the cerebral sickle **(B)**.

An 8-year-old girl was admitted on July 6th, 2021. Four days before admission, the child had a body temperature of 39.2°C with headache and malaise. Three days before admission, she had symptoms of right-sided slanting of the mouth, slurred speech, right-sided limb weakness, and unstable holding. A head CT performed at another hospital showed a hypodense shadow next to the posterior horn of the right lateral ventricle (Figure 1A). She was given ceftriaxone anti-infective treatment for 2 days, but the symptoms did not improve. Therefore, she was transferred to our department.

**FIGURE 2:**
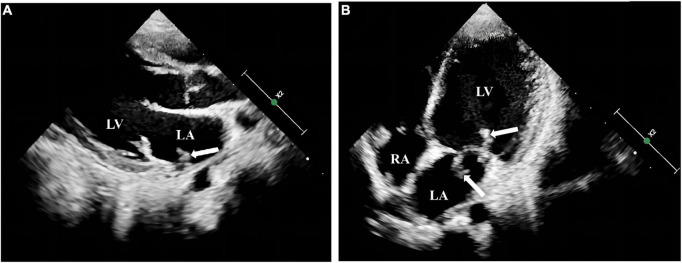
Transthoracic echocardiography examination of the heart in the pediatric patient. A striated flocculent hyperechoic attachment **(A)**, was observed at the entrance to the left pulmonary vein in the lateral posterior wall of the left atrium, with thickened anterior and posterior mitral leaflets and a striated flocculent hyperechoic attachment with a floppy texture visible in both anterior and posterior leaflets **(B)**. LA: left atrium; LV left ventricle; RA: right atrium.

**FIGURE 3:**
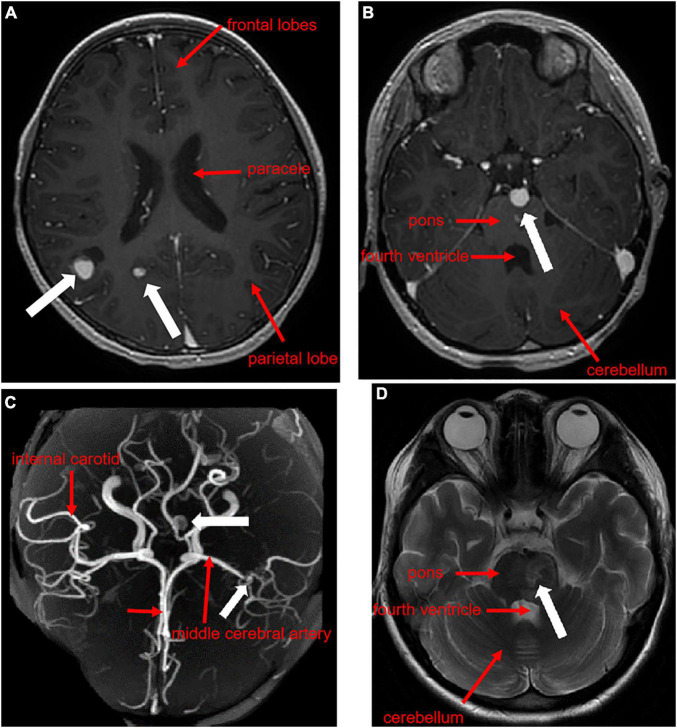
Contrast-enhanced cranial MRI + MRA of the pediatric patient. The transverse plane through the inferior portion of the parieto-occipital sulcus showed a distal aneurysm of the right middle temporal artery and the right posterior cerebral artery **(A)**, the transverse plane through the superior cerebellar peduncle showed an aneurysm at the tip of the basilar artery and stenosis of the left middle cerebral artery with multiple collateral vessel formation **(B,C)**, and the transverse plane through the superior cerebellar peduncle showed an abdominal cerebral infarction **(D)**.

Physical examination showed mild anemia, pharyngeal congestion, enlarged type II tonsils with yellowish-white secretions adhering to the surface, a heart rate of 116 beats per minute, and a class II/6 systolic murmur audible in the precordial region, neck stiffness, right-sided angle of the mouth, tongue extension to the right, grade IV muscle strength in the right limb, grade V muscle strength in the left limb, hyperalgesia on the right side, and positive Barthel’s sign on the right side. Auxiliary examination showed a routine blood count of 6.88 × 10^9^/L (reference range 5–12), neutrophils of 3.35 × 10^9^/L (reference range 2–7.7), and lymphocyte of 2.72 × 10^9^/L (reference range 0.8–4). Erythrocyte sedimentation rate was 100 mm/h (reference range 0–20), C-reactive protein of 40.67 mg/L (reference range 0–3), and procalcitonin of 0.05 ng/ml (reference range 0–0.05). Gram-negative bacteria lipopolysaccharide was negative. Tests of the cerebrospinal fluid (CSF) sample showed a white blood cell count of 17 × 10^6^/L (reference range 0–15), Mononuclear cell (MNC) of 16 × 10^6^/L, polymorphonuclear (PMN) of 1 × 10^6^/L, Cl of 126.1 mmol/L (reference range 117–127), glucose (GLU) of 2.37 mmol/L (synchronous peripheral blood glucose: 6.5 mmol/L), and protein quantification of 384.7 mg/L (reference range 100–200). CSF culture was negative. Transthoracic echocardiography showed thickening of the anterior mitral valve leaflet with vegetation attachment and vegetation formation at the entrance to the pulmonary veins in the lateral posterior wall of the left atrium (Figure 2). Contrast-enhanced MRI + magnetic resonance angiography of the head showed aneurysm formation in the right middle temporal artery, distal right posterior cerebral artery and apical region of the basilar artery, cerebral bridge infarction, and stenosis of the left middle cerebral artery with multiple collateral vessel formation (Figure 3).

PACEseq metagenomic next-generation sequencing (mNGS) (Hugobiotech, Beijing, China) of blood detected *A. defectiva* with a specific sequence number of 161. mNGS of CSF also detected *A. defectiva* with the specific sequence number of 165. The results were also confirmed by blood culture.

Multiple pathogen detection tools detected *A. defectiva*, but no drug sensitivity tests were performed because our hospital does not have criteria for drug sensitivity for this organism. Meropenem (20 mg/kg, once every 8 h) combined with vancomycin (10 mg/kg, once every 6 h) was given empirically as anti-infective treatment, and the body temperature returned to normal. Half a month after the combination anti-infective treatment, the child showed no positive neurological signs. The repeated blood culture was negative, the blood and CSF mNGS were negative, and the antibiotics were adjusted to ceftriaxone combined with vancomycin for anti-infective treatment.

On the 36th day after hospitalization, the child developed a severe headache and slurred speech, which recovered with anti-infective treatment. A CT head examination was completed, which showed a right parietal hematoma and a falciform herniation of the brain (Figure 1B). A pseudoaneurysm hemorrhage was considered. She then immediately underwent right temporo-occipital hematoma removal, cerebral vascular malformation resection, and cranial decompression. After the surgery, she gradually regained her speech. After a total of 60 days of hospitalization, her family requested to be discharged. At this point, the child was conscious, her speech ability had returned to normal, only the right upper limb muscle strength was grade III (may be related to the posterior cone crossover damage in the child), the rest of the limb muscle strength was normal, and there was still valve redundancy on cardiac ultrasonography, which was unchanged from before.

## Discussion

Infective endocarditis (IE) caused by *A. defectiva* usually occurs in children with congenital heart disease or a history of previous cardiac surgery (7), but some patients have no underlying disease (6). However, oral surgery related to dentistry is the most common cause (8–10). In this case, the child had no congenital heart disease and no history of cardiac-related surgery, but had septic tonsillitis at the time of presentation, which may have contributed to this child’s IE.

*Abiotrophia defectiva* is slow-growing and nutritionally demanding, usually growing only on media containing L-cysteine or vitamin B6 (11). Gram staining results are variable and morphologically diverse, making culture and identification difficult and diagnosis easily missed or delayed (12), which may explain the negative blood cultures in pediatric patients with IE (8, 13). Compared to conventional culture, mNGS is more sensitive (14, 15) and is particularly suitable for the detection of mixed and rare infections (4, 16). mNGS has been widely used in the diagnosis of clinical infectious diseases (7, 17), and has been reported for the clinical diagnosis of IE (14, 18). In this case, *A. defectiva* was detected in blood and CSF specimens by mNGS within 48 h of admission, but only one blood culture at admission was positive, while other blood cultures and CSF cultures were negative, indicating that the sensitivity of mNGS is much higher than that of traditional culture methods and can be used as an important means for identification of pathogens in patients with IE. Although it needs to be acknowledged that the pathogen was also detected by culture, and that the isolated strain could be used for the assessment of the antibiotic susceptibility tests.

Previous studies have shown that *A. defectiva*-associated IE may be resistant to penicillin but sensitive to a variety of antibiotics, such as third-generation cephalosporins, meropenem, vancomycin, gentamicin, and linezolid (11, 19). Specifically, it has been suggested (14) that *A. defectiva* is 94.6% susceptible to ceftriaxone, 91.9% susceptible to clindamycin, and 100% susceptible to meropenem, imipenem, vancomycin, and levofloxacin. Others (20) suggested that *A. defectiva* is 8, 92, and 83% susceptible to penicillin, amoxicillin, and ceftriaxone, and 100% susceptible to meropenem, clindamycin, rifampicin, levofloxacin, ofloxacin, and vancomycin, respectively. A combination of drugs is recommended. Due to the fact that there was no previous experience in the anti-infective treatment of this organism in our hospital, we chose meropenem combined with vancomycin based on the literature. As the child’s condition improved, we replaced meropenem with ceftriaxone to prevent bacterial resistance and secondary infection by other pathogens caused by prolonged use of broad-spectrum antibiotics. Because ceftriaxone can also cross the blood–brain barrier, it is also useful for central nervous system infections.

Compared to IE caused by other pathogens, *A. defectiva*-associated IE is more likely to form valvular vegetation and produce extracardiac organ embolic complications (21, 22), with poor prognosis and high mortality. The most common complication of the heart in IE is the formation of valvular redundancy, with mitral valve involvement being the most common, followed by aortic valve, and in rare patients, aortic, tricuspid, and pulmonary valves (8, 12, 14, 23), which can even be complicated by valve perforation in some cases (24). Extracardiac organ embolism can cause cerebral infarction, intracranial aneurysms, splenic infarction, infectious pneumonia, limb arterial embolism, aneurysms, etc. (12, 21, 25). On admission, the child was found to have vegetation on the mitral valve. The subsequent review revealed that vegetation was also observed at the entrance to the pulmonary veins of the left atrial and combined with multiple intracranial pseudoaneurysm formation, which is rare in clinical practice. Such a child usually requires surgical intervention. However, this child had both heart valve vegetation and multiple intracranial pseudoaneurysms, which are extremely risky for clinical procedures and may lead to an incurable and very poor prognosis.

In summary, *A. defectiva* is a rare pathogen causing IE, and the possibility of this disease should be considered especially in patients with IE having negative blood cultures. Because of the harsh culture conditions and low positivity rate of *A. defectiva*, mNGS is worthy of clinical application.

## Data Availability Statement

The original contributions presented in this study are included in this article/supplementary material, further inquiries can be directed to the corresponding author.

## Author Contributions

YD and YW conceptualized and designed the study, drafted the initial manuscript, and reviewed and revised the manuscript. ZZ, CC, HZ, and ZG collected data, carried out the initial analyses, and reviewed and revised the manuscript. HX designed the data collection instruments, coordinated and supervised data collection, and critically reviewed the manuscript. All authors approved the final manuscript as submitted and agreed to be accountable for all aspects of the work.

## Conflict of Interest

HX was employed by Hugobiotech Co., Ltd. The remaining authors declare that the research was conducted in the absence of any commercial or financial relationships that could be construed as a potential conflict of interest.

## Publisher’s Note

All claims expressed in this article are solely those of the authors and do not necessarily represent those of their affiliated organizations, or those of the publisher, the editors and the reviewers. Any product that may be evaluated in this article, or claim that may be made by its manufacturer, is not guaranteed or endorsed by the publisher.
